# Biological effects and regulation of IGFBP5 in breast cancer

**DOI:** 10.3389/fendo.2022.983793

**Published:** 2022-08-25

**Authors:** Jürgen Dittmer

**Affiliations:** Clinic for Gynecology, Martin Luther University Halle-Wittenberg, Haale (Saale), Germany

**Keywords:** insulin-like growth factor binding protein 5, breast cancer, estrogen, transcriptional regulation, breast cancer susceptibility region

## Abstract

The insulin-like growth factor receptor (IGF1R) pathway plays an important role in cancer progression. In breast cancer, the IGF1R pathway is linked to estrogen-dependent signaling. Regulation of IGF1R activity is complex and involves the actions of its ligands IGF1 and IGF2 and those of IGF-binding proteins (IGFBPs). Six IGFBPs are known that share the ability to form complexes with the IGFs, by which they control the bioavailability of these ligands. Besides, each of the IGFBPs have specific features. In this review, the focus lies on the biological effects and regulation of IGFBP5 in breast cancer. In breast cancer, estrogen is a critical regulator of IGFBP5 transcription. It exerts its effect through an intergenic enhancer loop that is part of the chromosomal breast cancer susceptibility region 2q35. The biological effects of IGFBP5 depend upon the cellular context. By inhibiting or promoting IGF1R signaling, IGFBP5 can either act as a tumor suppressor or promoter. Additionally, IGFBP5 possesses IGF-independent activities, which contribute to the complexity by which IGFBP5 interferes with cancer cell behavior.

## Introduction

Breast cancer (BC) is the most frequent cancer among women and leading cause of cancer-related death in women worldwide (1). As a systemic disease, it is characterized by early tumor cell dissemination, colonization of other organs and metastasis formation (2–4). It is generally accepted that BC is a cancer stem cell (CSC) disease, where CSCs drive cancer initiation, progression and metastasis (5, 6). Epithelial, quasi-mesenchymal and mesenchymal forms of CSCs have been identified and seem to have different functions in BC progression (7, 8).

BC is a heterogenous disease requiring subtype assessment to offer patients appropriate treatments. Classically, subtyping is performed by immunohistochemical examination of the expression of estrogen receptor α (ERα), progesterone receptor (PR) and the human epidermal growth factor receptor 2 (Her2). Approximately two-thirds of the BCs are positive for ERα. ERα expression is indicative of potential responsiveness of the cancer to ERα-targeting drugs (endocrine therapy), such as tamoxifen (TAM), fulvestrant (FULV), and aromatase inhibitors (9–11). Her2 overexpression, caused by amplification of its gene *erbb2*, defines a second major subtype (12). This subtype is potentially responsive to anti-Her2 antibodies or Her2 kinase inhibitors (13). A third subtype, triple-negative BC (TNBC), is devoid of ERα, PR- and Her2 and is routinely treated by chemotherapy (14). Molecular subtyping, based on mRNA profiling, revealed a heterogeneity of BCs beyond ERα, PR and Her2 expression, giving rise to the distinction of luminal A, luminal B, Her2-enriched and basal-like BCs (15). Clinically, the distinction between luminal A and the more aggressive luminal B, both predominantly ERα-positive BCs, is important (16).

In ERα-positive BCs, the insulin-like growth factor (IGF)/IGF1 receptor (IGF1R) pathway plays an important role in ERα-dependent regulation (17–19). IGF bioavailability and activity is controlled by IGF binding proteins (IGFBPs) (20–22). Hence, their abundances are crucial for the activity of the IGF/IGF1R pathway and, consequently, for ERα activity. Six IGFBPs are known (22). Of these, IGFBP5 may be of particular importance for IGF regulation in BC, as its gene is not only located in the vicinity of the BC susceptibility locus q35 on chromosome 2, but its activity also regulated by enhancer elements within this region (23–27). Moreover, ERα is a major driver of IGFBP5 transcription. Besides IGF-dependent effects, IGFBP5 shows also IGF-independent effects, which interferes with the behavior of BC cells (22).

This review intends to summarize the current knowledge on the biological effects of IGFBP5 in BC and on the regulation of its expression.

## Estrogen receptor α

The transcription factor (TF) ERα is classically activated by estrogen (28). ERα binds directly to estrogen-responsive elements or indirectly to other DNA sites by tethering to TFs, such as activating protein-1 (AP-1) (29, 30). It regulates a plethora of genes through proximal promoters and/or distal enhancers (31, 32). The interaction with the transcriptional machinery is mediated by two domains, transactivation function (AF)-1 and the estrogen-regulated AF-2 (33). Typically, ERα cooperates with the pioneer TF Forkhead box protein A1 (FoxA1), which facilitates ERα chromatin binding (34). Phosphorylation has a critical impact on ERα activity (33). Phosphorylations at Ser-118 and Ser-167 within the AF-1 domain promote estrogen-dependent as well as estrogen-independent transcriptional activities of ERα. Such phosphorylations can be triggered by receptor tyrosine kinases (RTKs) through the PI3K/AKT/mTOR/p70S6K and the Ras/Raf/MEK1/ERK1/2 pathways. Endocrine resistance, the resistance to endocrine therapy, is often caused by permanent activation of RTKs (35–37). Of the RTKs, IGF1R is of particular importance for ERα-dependent transcription.

## Involvement of the IGF/IGF1R pathway in the regulation of estrogen-dependent effects

Classically, insulin-like growth factor 1 receptor (IGF1R) either homodimerizes or forms heterodimers with the insulin receptor (IR) (38, 39). Under certain conditions, it can also heterodimerize with members of the Her RTK family (40). IGF1R activity is regulated by its ligands IGF1 and IGF2 and additionally modulated by six IGF binding proteins (IGFBPs) (20–22). By binding to IGF1 and IGF2, IGFBPs regulate their bioavailabilities. A tight control of IGF bioavailability is necessary, as, for instance, increased serum levels of liver-produced IGF1 correlates with higher risk of developing BC and other cancers (41). Like other RTKs, IGF1R activates the PI3K/AKT and Ras/Raf/MEK/ERK1/2 pathways, but involves the adapter proteins insulin receptor substrate 1/2 (IRS1/2) (17, 18, 42).

The IGF/IGF1R pathway modulates estrogen effects and ERα activity (17–19). IGF promotes ERα activity by stimulating ERα phosphorylation at Ser-167 (43). In turn, estrogen-activated ERα stimulates IGF1R gene transcription (17, 44, 45) and stabilizes IRS1 (46). ERα-independent effects of estrogen are executed by G-protein coupled receptor 30 (GPR30) (47). Activated by estrogen, GPR30 stimulates AKT and ERK1/2 phosphorylation (48–50). IGF1 and insulin stimulate the expression of GPR30 by involving a PKCδ/ERK1/c-Fos/AP-1 pathway (51, 52).

In primary BC, IGF1R is frequently expressed in luminal tumors (~50%) (53), likely reflecting its link to ERα signaling. In contrast, in the Her2-enriched or basal-like subtypes, only ~10% or ~20% of the tumors are positive for IGF1R, respectively. In luminal B tumors, higher IGF1R levels are associated with better prognosis, while, in luminal A tumors, IGF1R levels did not correlate with patients’ outcome (53). Its potential role in endocrine resistance has nevertheless stimulated research towards ways of targeting the IGF/IGFR signaling pathway (54).

## IGFBP5 as a major regulator of the IGF/IGF1R pathway

**Figure 1:**
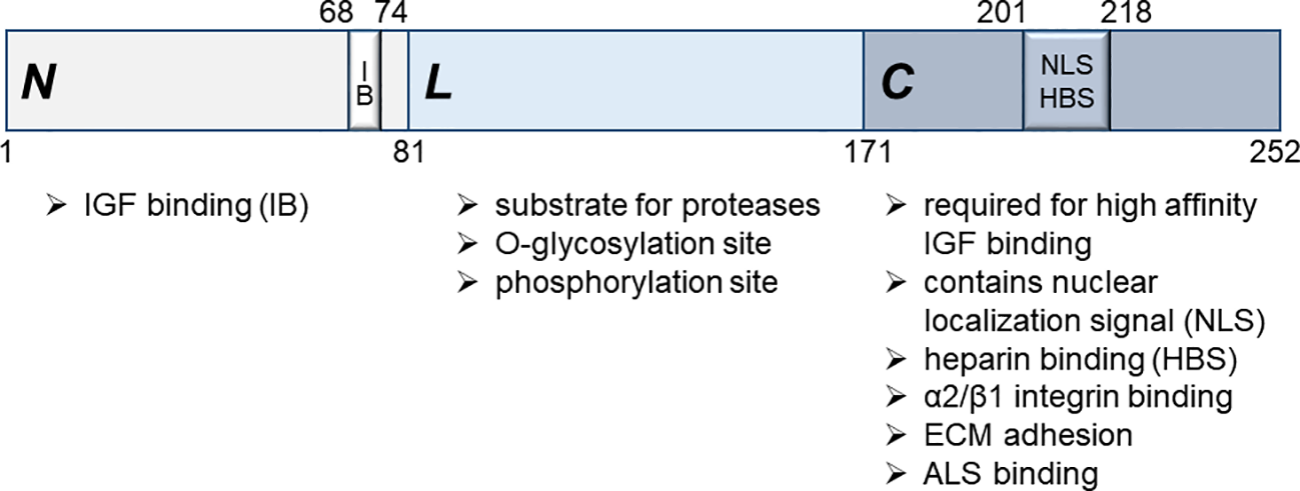
Structure of the human mature IGFBP5 protein. Three major domains, the N-terminal **(N)**, the linker **(L)** and the C-terminal **(C)** domain, can be distinguished, which fulfill different functions as indicated. For high affinity IGF binding, the C-terminal domain is required in addition to the primary IGF binding site (IB) within the N-terminal domain between aa 68 and 74. A nuclear localization signal (NLS) is located within the C-terminal domain between aa 201 and 218, which also mediates binding of the IGFBP5 protein to heparin.

IGFBPs consist of three distinct domains, the cysteine-rich N-terminal, the C-terminal and the linker domain (L-domain) (55). Of the six IGFBP genes, IGFBP5 is the most conserved one (55), whose cDNA was first isolated in 1991 (56, 57). The mature IGFBP5 protein shows an apparent molecular weight of 31-32kD (58, 59) and consists of 252 amino acids (60), of which aa 1-80 represent the N-terminal, 81-170 the L- and 171-252 the C-terminal domain (61) (**Figure 1**). The L-domain serves as a substrate for various proteases, such as ADAM9, MMP-2, PSA, thrombin and PAPP-A, and can be O-glycosylated and phosphorylated (62–66). A fragment of IGFBP5 encompassing amino acids 40-92 within the N-terminal domain is sufficient for IGF1 binding (21). The interaction is predominantly based on hydrophobic contacts, whereby Val-49, Leu-70 and Leu-73 of the IGFBP5 protein play a crucial role (60). Hence, mutations between amino acids 68 and 74, including Leu-70 and Leu-73, abolishes IGF binding (59, 67). However, for efficient inhibitory action of IGFBP5 on IGF1-IGF1R binding, the C-terminus of IGFBP5 is also important (60). Consistent with this finding, a 23kD truncated version (aa 1-169), lacking the C-terminal domain, binds to IGF with lower affinity (68, 69). Mutational analyses confirmed the participation of the C-terminal domain in IGF binding (70, 71). Particularly, Gly-203 and Gln-209, which are part of the C-terminal heparin-binding domain (see below), are involved in IGF1/IGFBP5 interaction. In blood, IGF and IGFBP5 form a ternary complex with acid labile subunit (ALS), which binds to the C-terminal domain of IGFBP5 (72).

**Figure 2:**
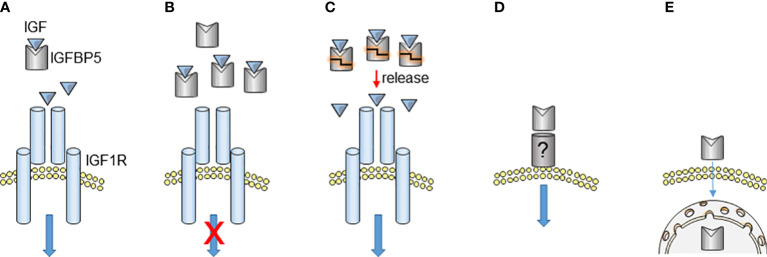
IGFBP5 actions. **(A, B)** By forming an inhibitory complex with IGF, IGFBP5 can prevent IGF-induced activation of IGF1R at appropriate IGFBP5/IGF ratios. **(C)** IGF-bound IGFBP5 has also been suggested to function as an IGF storage complex. Sudden release of IGF due to IGFBP5 proteolysis can strongly increase the local IGF concentration, potentially enhancing the IGF effect on IGF1R. In this scenario, IGFBP5 acts as a promoter of IGF activity. **(D, E)** IGF-independent effects of IGFBP5 either caused by its interaction with a putative plasma membrane receptor **(D)** or caused by its ability to enter the cell and accumulate in the nucleus **(E)**.

Although IGFBP5 exerts an inhibitory effect on the IGF/IGFR pathway by binding to IGF, a supportive effect of IGFBP5 on this pathway has also been reported (**Figure 2**). Recombinant IGFBP5 and IGF1 proteins together acted protective against ceramide-induced apoptosis of MCF-7 BC cells (73) and stimulated proliferation of human intestinal smooth muscle cells (74). In prostate cancer, where androgen deprivation causes prostate carcinoma and stromal cells to upregulate the IGFBP5 level (75, 76), overexpression of IGFBP5 enhances the anti-apoptotic and mitogenic activity of recombinant IGF1 protein, thereby helping to overcome castration-induced tumor regression (77, 78). Also, IGFBP5 enhances the anti-apoptotic effect of IGF on osteosarcoma cells (72). It has been proposed that, for the IGFBP5-promoting effect on IGF1, IGFBP5 presents and releases IGF1 to the receptor (73). Proteolysis of IGFBP5 may play a role in this process, in a way that IGFBP5 digestion by enzymes, like L-domain cutting PAPP-A, leads to a release of IGFBP5-bound IGF1 (72). Hence, not only is IGFBP5 an IGF inhibitor, but also seems to function as a reservoir for IGF, ready to release this growth factor, when required.

Based on data obtained with human intestinal smooth muscle cells, IGFBP5 may also indirectly promote IGF1 activity (79). IGFBP5 was shown to stimulate the secretion of IGF1 in an IGF-independent manner by activating ERK1/2 and stress-responsive p38 kinase. In turn, IGF1 promotes the expression of IGFBP5 to further fuel IGF1 production.

## IGF-independent activities of IGFBP5

IGFBP5 can exert effects independently of its IGF-interfering activity. Such IGF-independent effects may be executed intracellularly (intrinsic effects) or may involve IGFBP5 binding to a putative plasma membrane receptor (**Figure 2**). First indications for IGF-independent effects were provided by data showing that the IGFBP5 protein binds to the plasma membrane of osteoblasts and mesangial cells and is taken up by these cells (68, 69, 80, 81). The attachment to the plasma membrane can occur in the presence or the absence of the C-terminal domain, whereby the C-terminal domain contains a heparin-binding domain between aa 201-218, which allows membrane binding through heparin. The heparin-binding domain was also shown to be involved in IGF1-binding of the C-terminal domain, whereby it seems that IGF1 and heparin compete for binding to this site (71). The heparin-binding IGFBP5 (aa 201-218) fragment is able to stimulate migration of mesangial cells (82), while an IGFBP5 version lacking the C-terminus can increase proliferation of osteoblasts in an IGF-independent manner (69). Furthermore, the C-terminal domain shows biological activities that are distinct from that of the N-terminal domain (61).

Both IGF-dependent and IGF-independent effects were also detectable in transgenic mice. When overexpressed, wildtype IGFBP5, but not mutant IGFBP5 lacking a functional IGF-binding site was able to rescue embryonic development defects as induced by IGF2R loss (59) indicating, that for this effect, IGF binding was necessary. In contrast, overexpression of either IGFBP5 version caused a reduction in birth weight suggesting that IGFBP5-induced growth retardation did not rely on IGF-binding.

Part of the intrinsic effect of IGFBP5 seems to be based on its ability to enter the nucleus (83). The C-terminal domain contains a functional nuclear localization sequence (NLS, aa 201-218), which is identical to the heparin binding site (84–86). It interacts with importin β and the nucleolar protein nucleolin. Strikingly, an NLS-mutated version of IGFBP5 is able to fully support the IGF-induced anti-apoptotic effect on osteosarcoma cells (72), suggesting that the nuclear effects of IGFBP5 are independent of the IGF-depending ones.

One intracellular target of IGFBP5 is the Ras-Association Domain Family 1 C Protein (RASSF1C) (87). RASSF1C is a proto-oncogenic protein (88) that participates in driving invasion, metastasis and stem cell activity in BC (89). By physically interacting with RASSF1C, IGFBP5 may regulate ERK1/2 activity (87). Blocking the activity of RASSF1C resulted in loss of IGFBP5’s ability to stimulate ERK1/2 activity in osteoblasts. In BC cells, IGFBP5-induced ERK1/2 activation results in protection against apoptosis (90). IGFBP5 may also inhibit ERK1/2 activity. In lung cancer, IGFBP5 counteracted RASSF1C-induced ERK1/2-dependent stimulation of the stem cell gene Piwi like RNA-mediated gene silencing 1 (PIWIL1) resulting in re-sensitization of tumor cells to the anti-cancer drug betulinic acid (91). In osteoblasts, IGFBP5 has been found to interact with the heterodimer consisting of the vitamin D receptor and retinoid X receptor, thereby blocking vitamin D-dependent gene expression (92). IGFBP5 has also been shown to bind to Four and a Half LIM protein 2 (FHL2), though the significance of this interaction is not known yet (93).

IGF1-independent IGFBP5 effects based on extracellular interactions involve plasma membrane proteins, such as tumor necrosis factor receptor 1 (TNF1R), α2/β1 integrins and a 420 kD complex harboring a serine/threonine kinase (94–96). By binding of IGFBP5 to TNF1R, which requires its N- and L-domains (94), IGFBP5 interferes with TNFα activity. As shown with lymphoma cells, IGFBP5 blocks TNFα-dependent NFκB activation (94). *Vice versa*, TNFα prevents the L-domain-dependent interaction between IGFBP5 and TNF1R (94) and inhibits IGFBP5-dependent myoblast survival (97). On the other hand, IGFBP5 can also support TNFα activity and increases its NFκB-dependent pro-apoptotic action on MDA-MB-231 BC cells (98). In the latter case, however, it is not clear whether this effect of IGFBP5 involves its interaction with TNF1R.

The interaction of IGFBP5 to α2/β1 integrins causes MCF-7 cells to stronger adhere to the extracellular matrix (95). This effect involves activation of the GTPase Cdc42 and AKT as well as inactivation of the stress-response kinase p38. Interestingly, Cdc42 was also shown to be activated in the migration-stimulatory effect of the heparin-binding IGFBP5 fragment (aa 201-218) on mesangial cells (82).

A 420 kD plasma membrane complex had been identified to interact with IGFBP5 (96). Full length IGFBP5, IGFBP5 (aa 1-169) lacking the C-terminal domain and the IGFBP5 (201-218) harboring the heparin-binding domain were all found to be able to bind to this complex and to activate a serine/threonine kinase within this complex.

## Cellular source of IGFBP5

Cancer cells, including BC cells, express and secret IGFBP5 (99–101). In low-grade invasive BC, ~50% of the cancer cells show well detectable protein levels of IGFBP5 in the cytoplasm (102). IGFBP5 is also found in the nucleus of BC cells (103). The expression level of IGFBP5 is lower in normal breast, in normal tissue adjacent to the cancer lesion and in benign breast lesions (102, 104–106).

The microenvironment may also contribute to IGFBP5 production in cancer. Particularly fibroblasts, which in cancers are found in a modified form, called cancer-associated fibroblasts (CAFs) (107, 108), are a major source of secreted IGFBP5 protein in normal tissues (109–111). Attached to extracellular matrix (ECM) proteins, such as collagen, fibronectin and laminin, IGFBP5 is protected against proteolytic cleavage and can exert its effects on other cells. IGFBP5 can also feed back on fibroblasts by stimulating these cells to produce more ECM proteins, such as collagen 1A1 (109). Interestingly, in BC specimens, collagen 1A1 mRNA levels correlate with those of IGFBP5 (112). Excessive secretion of IGFBP5 by fibroblasts can block epithelial cell proliferation after injury, induce fibrosis and lead to premature senescence of fibroblasts (86, 109, 113–115). Senescent fibroblasts can acquire a senescence-associated secretory phenotype which can promote tumor progression (116) indicating that IGFBP5 may be involved in the generation of CAFs. In prostate cancer, stromal cells, particularly fibroblasts, were found to be the major source of IGFBP5 (117, 118) suggesting that fibroblasts contribute to IGFBP5 production also in cancer.

## IGFBP5 as a potential prognostic/predictive biomarker in breast cancer

A Japanese study on a total of 167 BC patients revealed a correlation of higher IGFBP5 expression with poorer disease-free survival for patients with ERα-positive tumors, whereas no such correlation was found, when patients with ERα-negative tumors were included in the analysis (119). In contrast, a Chinese study on 108 patients showed that higher IGFBP5 mRNA levels were significantly associated with poorer disease-free survival for patients with ERα-negative tumors, but not for those with ERα-positive tumors (106). A third, US-American study on 76 patients showed a correlation of higher IGFBP5 protein expression with worse metastasis-, relapse-free and overall survival irrespective of the ERα status (104). An *in silico* study revealed that higher IGFBP5 RNA was associated with poorer overall survival in luminal A, but not in luminal B cancer (90). In sum, all these studies show that higher IGFBP5 expression indicates poorer prognosis, though the studies remain inconclusive in regard to the role of the ERα status. It is unclear if the correlation between IGFBP5 and prognosis is coincidential or causitive in nature. IGFBP5 shows diverse effects on apoptosis, cell-ECM attachment and cellular migration (see below).

Interestingly, under certain treatment conditions, higher IGFBP5 expression was associated with better outcome. Of the 153 TAM-treated BC patients with mostly lobular cancer, those with higher tumoral protein expression of IGFBP5 had a higher probability to survive (120). Also, a study on 371 patients revealed that patients who received a chemotherapy less likely relapsed, if the nuclear IGFBP5 protein level was higher (103). This was not the case for patients who were never chemotreated. Hence, higher IGFBP5 protein expression may predict better response to endocrine and chemo therapy. *In vitro* studies suggest a causative role of IGFBP5 in anti-estrogen response (see below).

## IGFBP5’s effects on apoptosis, adhesion and migration

The Wnt pathway is critically involved in mammary development (121). Its deregulation by the mouse mammary tumor virus (MMTV) causes BC in mice. Hence, inhibition of this pathway by frizzled 8 receptor-immunoglobulin G Fc fusion protein (Fzd8CRD) leads to tumor regression (122). Part of this regression is caused by IGFBP5. Upregulated by Fzd8CRD, IGFBP5 inhibits the pro-survival effects of the IGF/IGF1R pathway. An inverse relationship between Wnt pathway activity and IGFBP5 expression is also found during mammary gland involution (122). Here again IGFBP5 acts pro-apoptotically and prevents IGF-depending survival of epithelial cells (122–126). Consistent with these results, IGFBP5 knock-out mice showed delayed involution after weaning (127). Since the proliferative activity of the breast epithelium is linked to the risk of developing BC in mice (128), the anti-mitogenic activity of IGFBP5 on breast epithelium fits well with the finding that enhancer activities that potentially increase IGFBP5 transcription are associated with lower BC risk (25–27, 129).

The Wnt pathway is also important for survival of basal-like BCs/TNBCs (6), which have much in common with the MMTV-induced BCs (121). The Wnt pathway might be responsible for the low IGFBP5 expression in basal-like BCs/TNBCs compared to that in other BCs (122). When TNBC cell lines MDA-MB-231 and Hs578T were exposed to IGFBP5, cells underwent apoptosis (98, 130, 131). This pro-apoptotic activity of IGFBP5 is likely to be IGF-independent, as both cell lines do not have a functional IGF survival pathway (130).

However, under certain conditions, IGFBP5 can switch from a pro-apoptotic factor to an anti-apoptotic factor. In the presence of pro-apoptotic ceramide, IGFBP5 inhibits apoptosis of Hs578T cells by promoting protein kinase C-dependent conversion of ceramide to anti-apoptotic sphingosine-1-phosphate (73, 132).

Also ERα-positive MCF-7 cells show a link between IGFBP5 and apoptosis, though the data are not conclusive. One study shows that PI3K inhibition, which causes apoptosis in this cell line (133), leads to higher transcription of the IGFBP5 gene causing ERK1/2 activity to rise and the expression of pro-apoptotic Bim to decline to counteract apoptosis (90). In contrast, in our study, MCF-7 cells responded to IGFBP5 knock-down by upregulation of AKT activity and IGF1R expression suggesting a pro-apoptotic activity of IGFBP5 in these cells (134). Yet another study demonstrated that recombinant IGFBP5 protein had little effect on MCF-7 cell survival, unless cells were treated with ceramide, in which case it counteracted ceramide-induced apoptosis (73). The dosage of IGFBP5 (overexpression vs. loss) and potential differences in IGF involvement in these effects may explain the conflicting results. In fact, increasing the dose of recombinant IGFBP5 was shown to switch IGFBP5 from being an anti-apoptotic to a pro-apoptotic agent in the presence of ceramide (73). And, MCF-7 cells have a functional IGF/IGF1R pathway allowing IGF-dependent IGFBP5 effects to come into play in addition to its IGF-independent effects.

Recombinant IGFBP5 protein can increase cell-ECM interactions. This was found with MCF-7 cells and required the C-terminal domain and α2/β1 integrins (95). The effect coincided with reduced cell-cell contacts and lower migratory activity. In non-transformed mouse mammary gland (NMuMG) cells, IGFBP5-induced changes in cell-ECM attachment activity depended upon the cellular phenotype (103). In cells with epithelial phenotype, IGFBP5 enhanced both cell-ECM interaction and migratory activity, while, in mesenchymal cells, it decreased attachment to ECM and had no effect on migration. These results emphasize again the cell context-dependent behavior of IGFBP5.

## Role of IGFBP5 in anti-cancer drug response

**Figure 3:**
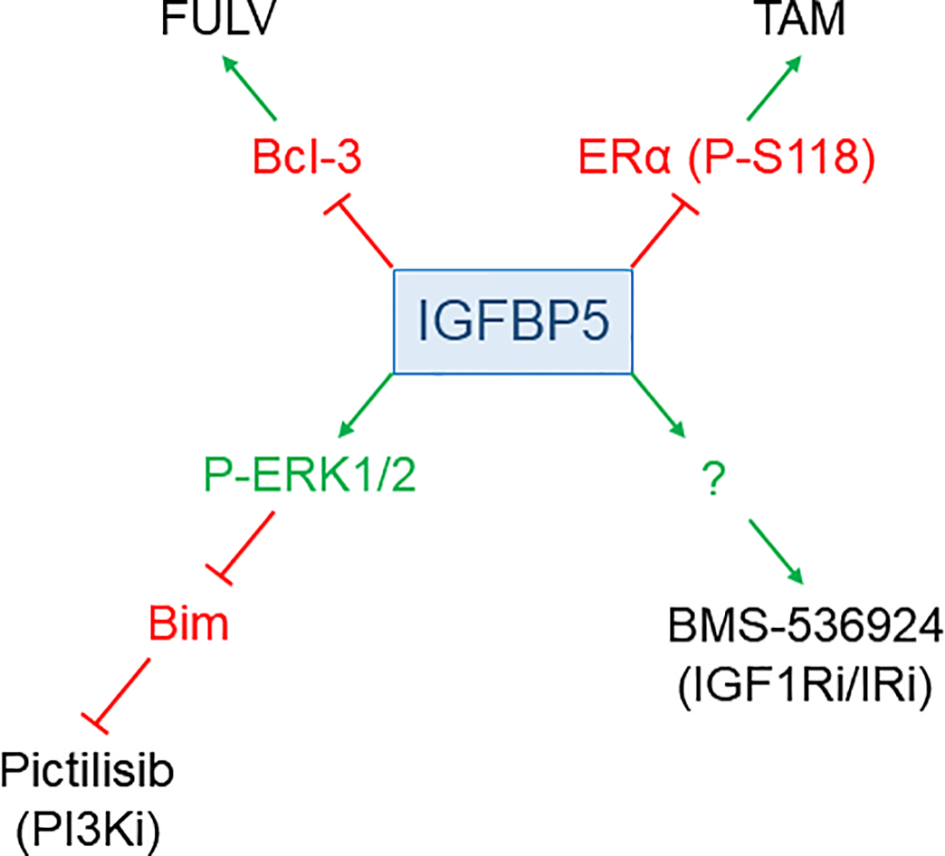
IGFBP5 modulates drug responses of BC cells. IGFBP5 counteracts resistance to the anti-estrogens FULV and TAM by either inhibiting the expression of Bcl-3 or by preventing phosphorylation of ERα at S118, respectively. On the other hand, IGFBP5 de-sensitizes cells to the PI3K inhibitor (i) GDC-0941, involving ERK1/2 activation and downregulation of pro-apoptotic Bim, and to the dual IGF1R/IR inhibitor BMS-536924 through a not yet defined mechanism.

Evidence has been accumulated that IGFBP5 plays a role in the responsiveness to anti-cancer drugs in BC (**Figure 3**). Depending on the anti-cancer drug, IGFBP5 either sensitizes or de-sensitizes BC to such drugs.

### Anti-estrogens

One frequent event leading to endocrine resistance is permanent activation of the PI3K/AKT signaling pathway by highly active RTKs (37). RTKs involved in endocrine resistance include Her family members, FGFR and IGF1R. The role of IGF1R in endocrine resistance is complicated as the IGF1R pathway is linked to ERα activity (see above). As estrogen stimulates IGF1R expression, FULV by inhibiting ERα has the opposite effect (45) and lower levels of IGF1R are found in FULV- and TAM-resistant MCF-7 sublines (135, 136). Nevertheless, the remaining IGF1R protein may still be important for growth of FULV-treated MCF-7 cells (136), as it could be hyperactivated by a FULV-induced rise in IGF1 expression (45) or by upregulation of the IGF2 level and simultaneous downregulation of the IGFBP5 level (134). However, a clinical study on the potential association of IGF1R expression and prognosis did not confirm a critical role of IGF1R in endocrine resistance (137). Nor did an IGF1R-targeting drug (ganitumab) improve patients’ outcome in an endocrine resistance setting (138).

IGFBP5 may play a role in anti-estrogen resistance independent of the IGF/IGF1R pathway. Overexpressed in MCF-7 cells, IGFBP5 was found to inhibit estrogen-dependent phosphorylation of the ERα protein at Ser-118 leading to a reduction in ERα-dependent growth (101). Though the IGF/IGF1R pathway is critically linked to ERα-activity, the inhibitory effects of IGFBP5 on ERα-activity did not rely on the IGF/IGF1R pathway. Importantly, Ser-118 phosphorylation was found to be indicative for TAM resistance in primary BC (139) and can be induced by TAM and FULV treatment (33). Hence, by blocking ERα phosphorylation at Ser-118, IGFBP5 may counteract anti-estrogen resistance.

Another mechanism by which IGFBP5 could inhibit the acquisition of anti-estrogen resistance is by keeping the expression of B-cell leukemia/lymphoma 3 (Bcl-3) at bay (134). This IκB-like protein, able to activate the NFκB survival pathway (140), shows higher expression in TAM-resistant BCs (134, 141). By upregulating Bcl-3 expression, CAFs or mesenchymal stem cells (MSCs), stromal cells known to be involved in drug resistance (108), de-sensitize MCF-7 and BT474 cells to FULV (134). The CAF/MSC-induced increase in the Bcl-3 level, which subsequently leads to FULV tolerance, could be mimicked by IGFBP5 knock-down and did not depend upon the PI3K/AKT pathway suggesting that IGFBP5 controls Bcl-3 expression in an IGF-independent manner.

Knock-down of IGFBP5 in MCF-7 cells was also shown to induce TAM resistance (120). Furthermore, TAM-resistant MCF-7 cells were found to express IGFBP5 at a lower level than parental cells and could be re-sensitized to TAM by exposure to recombinant IGFBP5 protein. Though another study confirmed the lower IGFBP5 level in TAM-resistant MCF-7 cells, it failed to show a causal link between IGFBP5 expression and TAM resistance (142). In sum, there is evidence that IGFBP5 can counteract the acquisition of anti-estrogen resistance in an IGF-independent manner, though more research is needed to confirm this hypothesis.

### PI3K and IGF1R inhibitors

Given the important role of the PI3K/AKT pathway in endocrine resistance, PI3K inhibitors are used to prolong survival of breast cancer patients who developed resistant tumors (143). Resistance of MCF-7 cells to the PI3K inhibitor GDC-0941 (Pictilisib) has been shown to be accompanied by increased expression of IGFBP5 (90). The GDC-0941-dependent upregulation of the IGFBP5 level is caused by the conversion of methylated to acetylated Lys-27 of the histone 3 protein resulting in increased IGFBP5 promoter activity, whereby lysine methylase KDM6B is a key enzyme in demethylating Lys-27. Therefore, inhibition of KDM6B or knock-down of IGFBP5 re-sensitized cells to GDC-0941. The protective effect of IGFBP5 was shown to involve the activation of ERK1/2, which, in turn, suppressed the expression of the pro-apoptotic protein Bim. Hence, though IGFBP5 sensitizes BC cells to anti-estrogens, it promotes resistance to PI3K inhibitors applied to fight anti-estrogen resistance. This is another example pointing to the dual nature of IGFBP5 in cancer cell biology.

Also resistance of MCF-7 cells to BMS-536924, a dual inhibitor of IGF1R and insulin receptor, coincides with higher levels of IGFBP5 (104). There is evidence for a causative role of IGFBP5 in the acquisition of this resistance, as its knock-down re-sensitized resistant cells to BMS-536924, while its overexpression in parental cells had the opposite effect. Also, in a panel of 93 cell lines, established from breast, colon and lung cancers, lower IGFBP5 levels were indicative for a strong response to the IGF1R-specific human monoclonal antibody figitumumab (144). Furthermore, in bladder cancer, higher resistance to the selective IGF1R kinase inhibitor AEW541 coincided with higher IGFBP5 levels and lower IGF1R activity (145).

In sum, these data not only suggest that tumorally expressed IGFBP5 may serve as a useful marker to assess responsiveness of cancer cells to IGF1R inhibitors, but also that IGFBP5 is causatively involved in the acquisition of resistance to these drugs.

## Regulation of IGFBP5 expression

**Figure 4:**
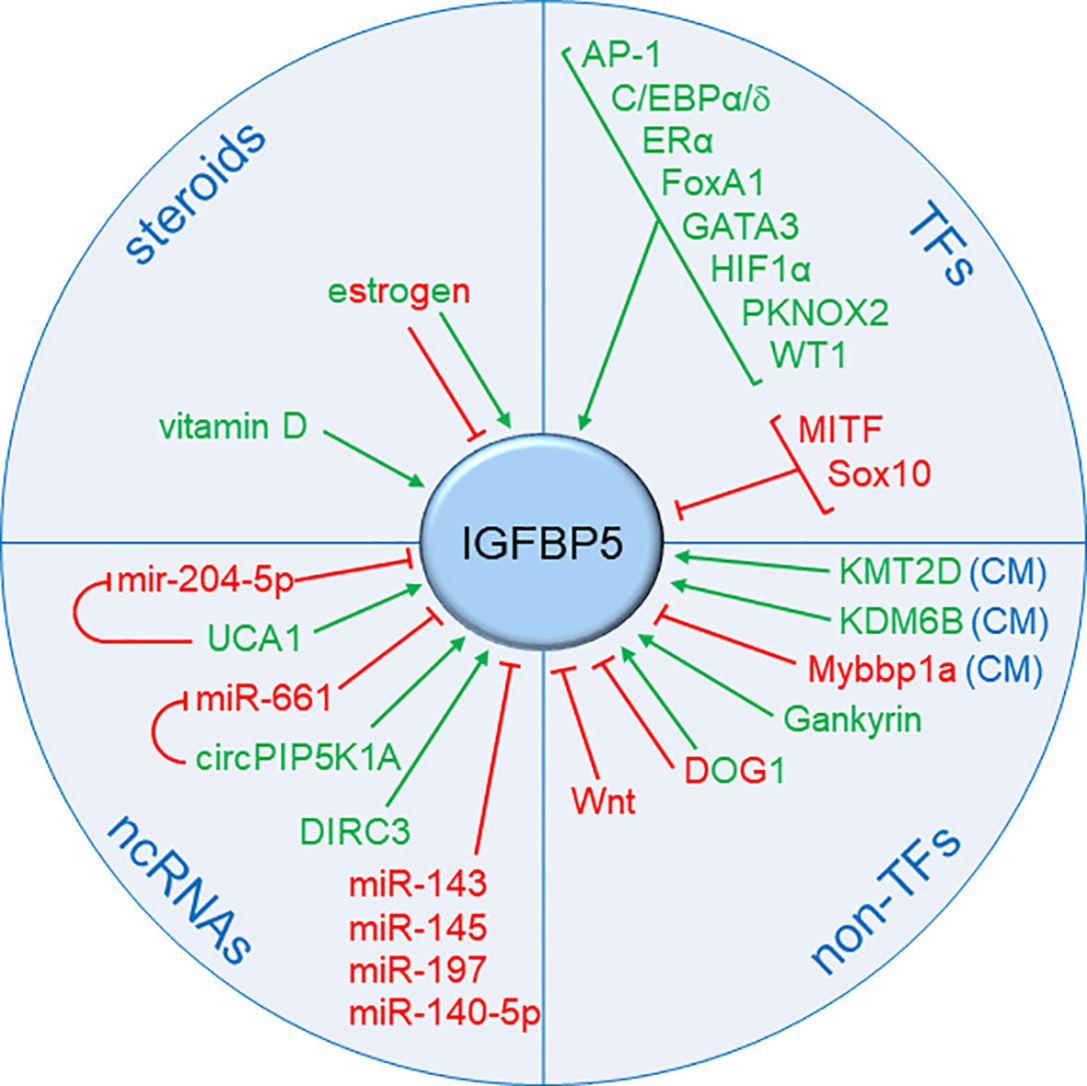
Summary of known factors regulating IGFBP5 expression. Besides proteins that regulate IGFBP5 transcription, namely transcription factors (TFs) (see also **Figure 5**) and chromatin modifiers (CM), other factors have been identified that interfere with IGFBP5 expression. These include other proteins (Wnt, DOG1, Gankyrin), steroids and non-coding RNAs (ncRNAs), namely microRNAs (miRs) and long noncoding RNAs (lncRNAs). Factors that stimulate IGFBP5 expression are denoted in green, those which have a negative effect are shown in red. Factors, which can have either effect dependent on the cellular context, are bicolored.

A number of TFs, non-TFs, non-coding RNAs as well as steroids have been identified to affect IGFBP5 expression (**Figure 4**). In BC, its regulation by estrogen is of particular importance, as it links IGFBP5 to BC risk.

### Transcriptional regulation

**Figure 5:**
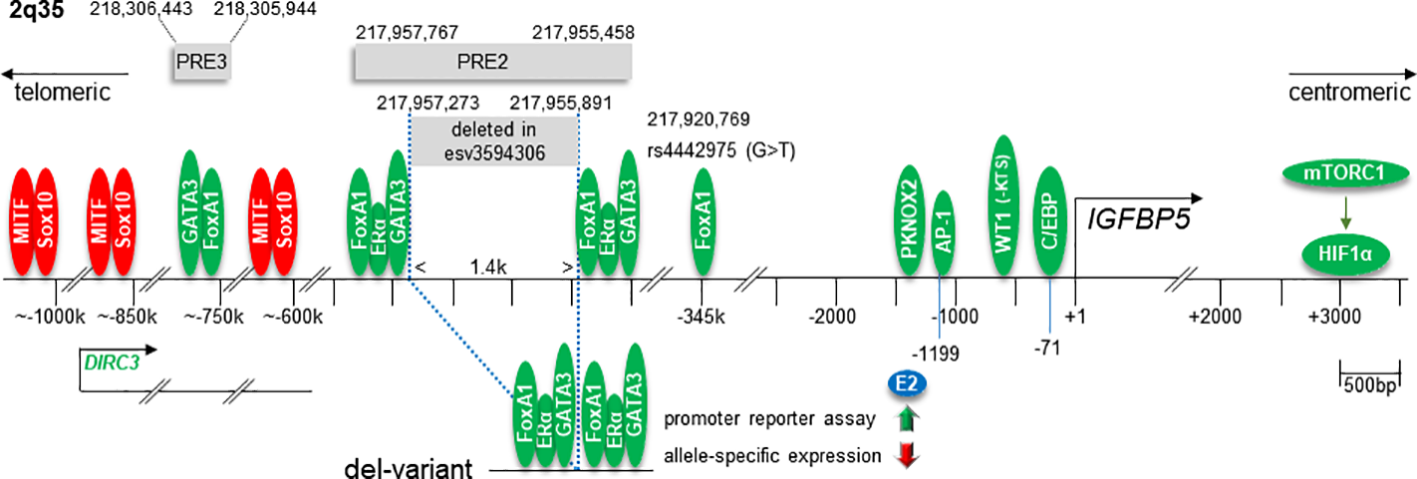
Overview of chromosome 2q regions relevant to the transcriptional regulation of the human IGFBP5 gene. These regions include the proximal IGFBP5 promoter, several enhancer elements in the protein-coding gene desert 2q35 and an HIF1α-binding downstream regulatory element. Numbers above or below the horizontal line indicate the genomic positions or the nucleotide positions relative to the transcriptional start site (+1) of the IGFBP5 gene, respectively. Green ovals indicate stimulatory transcription factors, red ones repressors. The relative position of the gene DIRC3, whose RNA product interferes with MITF and Sox10 activities, and the SNP site rs4442975, which interferes with FoxA1 binding activity, are indicated. In the lower panel, the transcription factor configuration in the deletion variant esv3594306 and its consequences for the estrogen (E2) response, as measured by promoter reporter or by allele-specific expression assays, are shown. PRE, putative regulatory element.

The IGFBP5 gene is located on the q-arm of chromosome 2 and is tightly linked to the IGFBP2 gene in a tail-to-tail orientation (146). Besides proximal promoter elements, sequences far upstream (telomeric) and downstream (centromeric) of the transcriptional start site (TSS) regulate IGFBP5 transcription (**Figure 5**). The far upstream regulatory elements are located in the 2q35 protein-coding gene desert (23–27). This region has been recognized as a BC susceptibility locus, where several single nucleotide polymorphism (SNP) sites and a deletion variant (called enCNV or esv3594306) have been identified, potentially linking IGFBP5 to BC risk (25–27, 129).

#### Factors binding to the breast cancer susceptibility locus 2q35

ERα is an important regulator of IGFBP5 transcription in ERα-positive BCs. Three independent studies on primary BCs revealed that ERα-positive BCs express higher IGFBP5 mRNA levels than ERα-negative BCs (119, 147, 148). Additionally, *in vitro* studies showed that estrogen modulates IGFBP5 expression in ERα-positive BC cell lines, whereby the mode of its action varies between cell lines (99, 147). In T47D and BT474 cells, estrogen upregulates (24, 147), whereas, in MCF-7 cells and in a TAM-resistant MCF-7 subline, it downregulates IGFBP5 expression (99, 149). In the latter cells, anti-estrogens TAM and FULV upregulate IGFBP5 expression instead. Additionally, MCF-7 cells show much lower IGFBP5 expression than T47D and BT474 cells (100) and harbor a monoallelic variant (esv3594306) within the chromosomal region 2q35, which is not found in the other two cell lines (see below). This variant may be responsible for the repressive effect of estrogen on IGFBP5 expression in MCF-7 cells (26). Of note, a repressive effect of estrogen on IGFBP5 was also found with the estrogen-sensitive PE04 ovarian cancer cell line (150).

Genome-wide association studies (GWASs) are used to identify low-penetrance genetic loci which are associated with diseases, such as cancer. Such studies have identified a BC susceptibility locus at 2q35 (151), which is hundreds of kilobase pairs (kbps) away from the IGFBP5 gene (23). Capture Hi-C, a method performed to map genomic contacts, revealed that proteins binding to 2q35 (~217,913 kbp- ~218,262 kbp) interact with proteins binding to the IGFBP5 gene (217,536,828 bp-217,560,272 bp) (23) suggesting a DNA loop being formed between 2q35 and the IGFBP5 gene. Subsequent studies identified several regulatory elements within the 2q35 region that interfere with the transcription of the IGFBP5 gene (23, 24, 26, 27, 152). One regulatory element, located ~345 kbp telomeric to the IGFBP5 promoter, is an enhancer that harbors a binding site for FoxA1 (23, 24). A SNP site (rs4442975, genomic position: 217,920,769 bp) has been identified at an important position of the FoxA1 recognition element. The replacement of the G at this site by a T significantly increases FoxA1 DNA binding. G/T heterozygous BC cell lines show higher IGFBP5 mRNA levels than G/G homozygous ones suggesting that the remote FoxA1-binding enhancer plays a role in IGFBP5 transcription. Studies on normal breast tissue confirmed the link between the T-allele of rs4442975 and higher IGFBP5 expression. Interestingly, estrogen responsiveness is also increased in the G/T heterozygous BT474 cells as compared to G/G homozygous MCF-7 cells. This finding may relate to the strong link between FoxA1 and ERα activities (34). Given this link, it is striking that the T-allele of rs4442975 is associated with a reduced risk of women to develop ERα-positive BC (27, 129). Moreover, the protective effect of the T-allele was stronger, when women were under current treatment with estrogen and progesterone, a therapy that increases the risk of developing ERα-positive BC by approximately two-fold.

In another 2q35 variant (esv3594306), a stretch of ~ 1.4 kbp between 217,955,891 and 217,957,273 is deleted, which has a profound effect on IGFBP5 transcription (26, 27). The deleted sequence is part of the putative regulatory element PRE2, located ~400kbp telomeric to the IGFBP5 gene. On both sites of the deleted sequence, functional binding sites for ERα and FoxA1 as well as for GATA binding protein 3 (GATA3), another ERα-cooperating TF (153), are found (27). Luciferase-based promoter assays showed that, by bringing the two composite enhancer elements in close proximity, the deletion potentiates the PRE2-dependent enhancer activity. Additionally, in an allele-specific chromosomal approach by using PRE2-heterozygous MCF-7 cells, a stronger binding activity of ERα to the deletion variant allele was found as compared to the WT allele (26). Interestingly, in the absence of estrogen, the variant allele was found to be the major driver of ERα-dependent IGFBP5 expression, whereas, in the presence of estrogen, IGFBP5 transcription on the variant allele was suppressed. This may explain the inhibitory effect of estrogen on IGFBP5 expression in MCF-7 cells (99, 149). Of note, in the luciferase-based promoter assay, estrogen did not suppress, but rather promote the activity of the deletion variant enhancer (27) suggesting that the inhibitory effect of estrogen on the variant allele is dependent upon the chromatin structure.

The esv3594306 deletion on one or both alleles is found in ~9% of European Americans and ~27% of African Americans (26). In both groups, carriers of this deletion have a reduced risk of developing BC. This association was stronger for ERα-positive than for ERα-negative BC supporting the notion that the ERα activity is important for the effect of esv3594306 on BC risk. Interestingly, evidence has been provided that this deletion does not affect the expression of IGFBP5’s neighboring genes IGFBP2 and RPL37A (27). This may suggest that changes in IGFBP5 expression is the major, if not only reason for the esv3594306-dependent reduction in BC risk. However, interchromosomal interactions of the 2q35 region have to be considered as well. A major interaction event in *trans* has been found between 2q35 and chromosome 1q42.12, which includes enable homolog gene (ENAH) (23). ENAH is frequently highly expressed in BC and shows an inverse expression to ERα suggesting that it is regulated by estrogen (154).

Even further upstream of the IGFBP5 gene an additional putatitive enhancer element, PRE3, has been identified (218,305,944- 218,306,443) (27). It contains functional GATA3 and FoxA1 sites and enhances IGFBP5 promoter activity in luciferase-based promoter assays with estrogen amplifying this effect.

Telomeric to PRE2 and including the PRE3 sequence lies a gene that codes for the long non-coding RNA (lncRNA) disrupted in renal carcinoma 3 (DIRC3) (152). DIRC3, which contains 3384 nucleotides, belongs to a subset of nuclear lncRNAs that control transcription of protein-coding genes. One target of DIRC3 is its neighbor IGFBP5. Two DNA looping interactions connect the DIRC3 locus to the IGFBP5 promoter. In melanoma cells, where DIRC3 is highly expressed, DIRC3 increases IGFBP5 transcription by preventing the TFs SRY-Box transcription factor 10 (Sox10) and melanocyte inducing transcription factor (MITF) from inhibiting IGFBP5 transcription. Sox10 is highly expressed in basal-like BCs (155) and might negatively regulate IGFBP5 transcription in this mostly ERα-negative subtype. Interestingly, Sox10 plays an important role in mammary development and a link between Sox10 and Wnt signaling has been suggested (156). It is therefore possible that Sox10 is involved in the inverse relationship between IGFBP5 expression and Wnt activity, as discussed above.

#### Factors binding to proximal promoter elements

Four functional TF recognition elements have been localized in the proximal human IGFBP5 promoter. These include binding sites for CCAAT-enhancer-binding proteins/C/EBPs), Wilms tumor gene 1 (WT1), activating protein-1 (AP-1) and PBX/Knotted Homeobox 2 (PKNOX2).

A C/EBP binding site has been identified in the proximal IGFBP5 promoter at position -71 relative to the transcriptional start site (TSS) (157, 158). In HNSCC, it allows C/EBPα to upregulate IGFBP5 expression (157). In primary rat osteoblasts, it mediates responsiveness to C/EBPδ and confers reactivity of these cells to prostaglandin E2 (158). C/EBPα is considered to act as a tumor suppressor in a number of solid cancers, including BC (159). In contrast, C/EBPδ shows tumor-promoting activities in BC, as evidenced by its ability to increase BC stem cell activity and to support BC growth and metastasis (160). Interestingly, in BC, expression of C/EBPα is upregulated by ERα and downregulated by HIF1α. On the other hand, C/EBPδ upregulates HIF1α expression in BC cells and, hence, might additionally promote IGFBP5 transcription indirectly through HIF1α (see below). Whether and to which extent C/EBP proteins contribute IGFBP5 expression in BC remains to be seen.

Further upstream of the C/EBP recognition element are several potential binding sites for WT1 between positions -848 and -190 relative to TSS (161). WT is a zinc finger TF, which may act as a tumor suppressor or promoter depending on the type of tumor (162). A short sequence (KTS) between the third and fourth zinc finger is important for its affinity to DNA. The splicing variant WT1(-KTS), which misses this sequence, was found to bind to and activate the IGFBP5 promoter in osteosarcoma cells (161). Interestingly, WT1 acts repressive on the IGF2 P3 promoter (163) suggesting that WT1 interferes with the balance between IGF2 and IGFBP5 expression. In BC, both isoforms, WT1 and WT1(KTS), are expressed and may play a role in ERα and Her2 expression (164).

A binding site for AP-1, hetero- or homodimeric TFs consisting of members of the Jun, Fos, ATF or Maf families (165), has been identified at position -1199 relative to IGFBP5 TSS (166). A SNP at -1195C>T, which reduced binding of AP-1 to the IGFBP5 promoter coincided with lower promoter activity in HNSCC cells. The T-allele was found to be associated with higher tumor grade in non-oropharyngeal HNSCC. In BC, AP-1 is well recognized as being involved in tumor progression (167). Additionally, it can serve as a docking protein for ERα. By tethering to AP-1, ERα is able to drive transcription through AP-1 binding sites (29). The AP-1 binding site within the proximal IGFBP5 promoter might therefore contribute to ERα-dependent IGFBP5 transcription.

Several recognition elements for PKNOX2, a homodomain TF, have been identified between -800 and -1800 relative to IGFBP5 TSS by using chromatin immunoprecipitation (ChiP) assays. PKNOX2 is able to upregulate IGFBP5 transcription in gastric cancer cells (168). Being a pro-apoptotic and anti-mitogenic protein, PKNOX2 acts tumor-suppressive on gastric cancer. There is evidence that IGFBP5 partially mediates this tumor-suppressive effect. Whether PKNOX2 plays a role in IGFBP5 expression in BC is not known.

#### HIF1α binding to a downstream recognition element

IGFBP5 was found to be a target of hypoxia-inducible factor 1α (HIF1α) (169). Expression of HIF1α is commonly regulated by hypoxia, but, particularly in cancer, can also be modulated by other factors, such as inhibitors of mammalian target of rapamycin (mTOR) (170). This important downstream target of the PI3K/AKT pathway forms two complexes, mTORC1 and mTORC2, of which mTORC1 negatively feeds back on this signaling pathway to limit the pathway’s activity. Part of this feedback mechanism includes secreted IGFBP5 (169). By stimulating the translation of HIF1α mTORC1 increases HIF1α protein abundance. In turn, HIF1α binds to multiple sites at a sequence approximately 3 kbp centromeric to the IGFBP5 promoter and increases its activity. Consequently, more IGFBP5 is secreted which then reduces IGF1R-dependent PI3K/AKT activity. Identified by using tuberous sclerosis complex (TSC)^-/-^ mouse embryonic fibroblasts, the IGFBP5-dependent feed back regulation of the PI3K/AKT pathway was also demonstrated to take place in cancer cells, including MCF-7 cells. Hence, the mTORC1/HIF1α/IGFBP5 axis may play a general role in restricting IGF1R-driven PI3K/AKT activity in (breast) cancer cells.

#### Chromatin modifiers

Chromatin accessibility is vital to active transcription. Post-transcriptional modifications of histones are key events in regulating chromatin accessibility (171). Certain histone modifications, such as H3K4me1, indicate open chromatin. In melanoma cells, the histone methyltransferase KMT2D, which can methylate Lys-4 in histone 3, has been found to keep proximal and distal IGFBP5-regulating enhancers in an open state, thereby contributing to high IGFBP5 levels (172). Both KMT2D and IGFBP5 act as tumor suppressors in melanoma cells and, in metastatic melanoma, the IGFBP5 level correlates with that of KMT2D. It is possible that KMT2D plays also an important role in ERα-dependent transcription of IGFBP5 in BC, as KMT2D is important for ERα/FoxA1-depending transcription (173).

Myb binding protein 1a (Mybbp1a) is involved in chromatin remodelling (174) and interferes with the function of certain nuclear factors (175). It acts primarily as a tumor suppressor. In Huh-7 human hepatocellular carcinoma (HCC) cells, Mybbp1a promotes the methylation of CpG islands within the IGFBP5 gene by binding to DNA methyltransferase 1 (DNMT1), thereby blocking IGFBP5 transcription (176). Expressed as well in BCs, Mybbp1a might regulate IGFBP5 gene methylation in BCs (177). Additionally, it might interfere with ERα-dependent transcription of IGFBP5. Mybbp1a has been shown to be an activating co-factor of estrogen-related receptor α (ERRα) (178), a TF whose recognition site overlaps with that of ERα (179). Interestingly, BC cells may rise the ERRα to ERα expression ratio in response to the ERα inhibitor TAM (37).

### Translational regulation by non-coding RNAs

Noncoding RNAs, including microRNAs (miRs) and lncRNAs, have an impact on IGF/IGF1R pathway activity (reviewed in (180)). MiRs are double-stranded short RNAs that are binding to the 3’-upstream region (UTR) of mRNAs to block their translation or to induce their degradation (181). A subset of lnc-RNAs, non-protein coding transcripts of sizes of more than 200 bases, are able to counteract the actions of miRs (182). Several miRs and counteracting lncRNAs were also found to be able to regulate IGFBP5 translation.

In breast tissues, miR-140-5p may be an important regulator of IGFBP5 translation (183). MiR-140-5p is more highly expressed in breast cancer lesions compared to normal breast tissue.

In papillary thyroid cancer (PTC), mir-204-5p was shown to suppress IGFBP5 expression and to inhibit cellular growth (184). Growth could be restored by overexpression of IGFBP5 or expression of the lncRNA Urothelial Carcinoma Associated 1 (UCA1) that limits the activity of miR-204-5p (185). In BC, miR-204-5p is often downregulated and considered to be a tumor suppressor (186), whereas UCA1 is overpressed and shows cancer-promoting effects (187, 188). Whether these activities in BC are linked to IGFBP5 has not been analyzed yet.

In ovarian cancer, the ratio between miR-661 and the circular lnc-RNA circPIP5K1A plays a role in regulating IGFBP5 translation (189). In the cell lines SKOV3 and A2780, circPIP5K1A and IGFBP5 promote cellular growth. In BC, miR-661 acts tumor-suppressive and higher miR-661 expression correlates with better outcome (190, 191). If miR-661 regulates IGFBP5 expression in BC is not known.

In meningioma cells, miR-197 was found to inhibit the expression of IGFBP5 (192). In BC, miR-197 seems to be upregulated compared to healthy breast tissue (193).

Other miRs that can potentially interfere with IGFBP5 translation are miR-143 and miR-145. In the intestine, these two miRs play a role in regulating IGFBP5 in injury-activated myofibroblasts (113). Like miR-204-5p in thyroid cancer cells (113), miR-143 was found to be counteracted by lncRNA UCA1 in colon cancer cells (194). A significant correlation between miR-143 and miR-145 expression was found in BC (195). Both were found to act as tumor-suppressors.

In sum, there are a number of miRs that potentially could interfere with IGFBP5 expression in BC. Of these, most act as tumor suppressors in BC. Only miR-197 and miR-140-5p might have tumor-promoting functions. It remains to be shown to what extent IGFBP5 contributes to their tumor-suppressing or tumor-promoting activities in BC.

### Regulation of IGFBP expression by other factors

The oncoprotein gankyrin is a subunit of the 26S proteasome and contributes to the degradation of proteins, such as p53 and Rb (196). Gankyrin has been shown to upregulate IGFBP5 expression in Huh-7 human hepatocellular carcinoma (HCC) and U-2 OS osteosarcoma cell lines (197). IGFBP5 knock-down in these cells resulted in growth inhibition. Gankyrin is also expressed in BCs, particularly in Her2-positive and TAM-resistant BCs (198, 199).

A calcium-dependent chloride channel protein called Discovered On Gastrointestinal stromal tumors 1 (DOG1) or anoctamin 1 (ANO1) (200) has a strong impact on IGFBP5 expression in gastrointestinal stromal tumor cell lines (201). In BC, DOG1 acts as a growth- and invasion-promoting factor, whose expression is associated with poorer survival (202).

Vitamin D-related compounds, which act through the vitamin D receptor, were also found to regulate IGFBP5 expression. Shown for MCF-7 cells, vitamin D increases the secretion of IGFBP5 and inhibit IGF-1-mediated cellular growth, an effect that was dependent upon the IGFBP binding domain of IGF-1 (203).

## Conclusions

On the one hand, higher IGFBP5 expression seems to predict lower risk of developing BC. On the other hand, higher IGFBP5 expression is linked to poorer outcome of BC patients. This apparent contradiction can be explained by the different functions of IGFBP5 in normal breast as compared to BC. In normal breast, it reduces the risk of BC initiation by counteracting mitogenic IGF and inducing apoptosis, while, in BC, it can have multiple functions, depending on BC subtype, the individual cellular context, the tumor microenvironment and on whether it acts IGF-dependent or -independent. The BC subtype is important, because IGF is a mitogen particularly in ERα-positive BCs and because ERα is a major driver of IGFBP5 transcription.

Given that IGFBP5 is a target of ERα, IGFBP5 could be expected to play a more specific role in ERα-positive BCs. However, no clear function has been defined yet even in this BC subtype. Data obtained from clinical studies are inconsistent as to whether IGFBP5 expression in ERα-positive BCs is of any value for predicting patients’ outcome as long as patients with any kind of ERα-positive BCs and treatment are evaluated together. Further specification by molecular taxonomy and/or by treatment may be necessary to define IGFBP5’s role(s) in ERα-positive BCs. So far, such more specific analyses has revealed a link between high IGFBP5 expression and poor survival of patients with luminal A, but not luminal B tumors, and an association between high IGFBP5 levels and more favorable outcome in TAM-treated patients with mostly lobular cancers. Only in the latter case are the clinical data backed up by *in vitro* experiments showing a sensitizing effect of IGFBP5 to anti-estrogens.

Given the mostly poor survival linked to higher IGFBP5 expression, it is puzzling that, in both ERα-positive and -negative BC cell lines, IGFBP5 shows pro-apoptotic activities. Hence, there must be additional, tumor-promoting IGFBP5 activities, which have yet to be uncovered. For instance, IGFBP5 might play a promoting role in BC cell dissemination and/or metastasis. In this case, IGFBP5 would have a dual effect in BC, suppressive in the initial phase by its pro-apoptotic activity and promoting in the late phase of tumor development by supporting the metastatic activity of the tumor. Such a behaviour would be reminiscent of the dual role of transforming growth factor β, which is known to act as a tumor suppressor in pre-malignant and as a tumor promoter in malignant cells (204).

Overall, it is not clear yet whether and how IGFBP5 is involved in BC progression. IGFBP5 might just be a surrogate marker and, as such, not contribute itself to BC progression. Clearly, more research is necessary to better understand the function of IGFBP5 in BC. Particularly, research focussing on IGFBP5’s role in BC dissemination, dormancy and metastatic outgrowth may advance our knowledge on the mechanism(s) by which IGFBP5 interferes with BC progression.

## Author contributions

The author confirms being the sole contributor of this work and has approved it for publication.

## Acknowledgments

The author thanks Angela Dittmer for critically reading the manuscript.

## Conflict of interest

The author declares that the research was conducted in the absence of any commercial or financial relationships that could be construed as a potential conflict of interest.

## Publisher’s note

All claims expressed in this article are solely those of the authors and do not necessarily represent those of their affiliated organizations, or those of the publisher, the editors and the reviewers. Any product that may be evaluated in this article, or claim that may be made by its manufacturer, is not guaranteed or endorsed by the publisher.
